# Submicroscopic aberrations of chromosome 16 in prenatal diagnosis

**DOI:** 10.1186/s13039-019-0448-y

**Published:** 2019-08-05

**Authors:** Xiaoqing Wu, Liangpu Xu, Ying Li, Na Lin, Linjuan Su, Meiying Cai, Xiaorui Xie, Lin Zheng, Hailong Huang, Yuan Lin

**Affiliations:** Fujian Key Laboratory for Prenatal Diagnosis and Birth Defect, Fujian Provincial Maternity and Children’s Hospital, affiliated Hospital of Fujian Medical University, No.18 Daoshan Road, Gulou District, Fuzhou, 350001 China

**Keywords:** Submicroscopic aberrations, Chromosome 16, Chromosomal microarray analysis, Prenatal diagnosis

## Abstract

**Background:**

Nearly 9.89% of chromosome 16 consists of segmental duplications, which makes it prone to non-homologous recombination. The present study aimed to investigate the incidence and perinatal characteristics of submicroscopic chromosome 16 aberrations in prenatal diagnosis.

**Results:**

A total of 2,414 consecutive fetuses that underwent prenatal chromosomal microarray analysis (CMA) between January 2016 and December 2018 were reviewed. Submicroscopic anomalies of chromosome 16 accounted for 11.1% (15/134) of all submicroscopic anomalies detected in fetuses with normal karyotype, which was larger than the percentage of anomalies in any other chromosome. The 15 submicroscopic anomalies of chromosome 16 were identified in 14 cases; 12 of them had ultrasound abnormalities. They were classified as pathogenic (*N* = 7), and variants of uncertain significance (*N* = 8). Seven fetuses with variants of uncertain significance were ended in live-born, and the remaining were end in pregnancy termination.

**Conclusion:**

Submicroscopic aberrations of chromosome 16 are frequent findings in prenatal diagnosis, which emphasize the challenge of genetic counseling and the value of CMA. Prenatal diagnosis should lead to long-term monitoring of children with such chromosomal abnormalities for better understanding of the phenotype of chromosome 16 microdeletion and microduplication syndromes.

## Background

It is well known that complete trisomy 16 is embryonic lethal, and it is the most common autosomal anomaly revealed by genetic diagnosis of spontaneous miscarriage [1]. Human chromosome 16 has one of the highest levels of segmental duplication: nearly 9% of genome-wide human duplication alignments map to this chromosome. Nearly 9.89% (7.8 Mb) of chromosome 16 consists of segmental duplications in the form of low copy repeats (LCRs) [2]. LCR16a, a 20 Kb low-copy repeat sequence, is the most frequent chromosome-specific duplication distributed in multiple locations over the entire length of chromosome 16 in a non-tandem manner. Most of it is concentrated on the short arm, including cytogenetic band locations 16p13.3, 16p13.1, 16p12.3, 16p12.2, 16p11, 16q22.2 and 16q23 [3]. This duplication may potentially lead to the rearrangement of the short arm segments of chromosome 16, which explains the high variability in breakpoints and sizes of 16p microdeletions and micro-duplications.

Although the segmental repeats are enriched in the relatively gene-poor pericentromere of the short arm, some are likely to have an impact on human disease susceptibility [2]. Several loci associated with chromosome 16 have been frequently examined in conjunction with susceptibility to disorders of the nervous system. In some studies concerning the utility of the chromosomal microarray analysis (CMA) in prenatal testing, microduplications or microdeletions associated with chromosome 16 were relatively frequent findings [4, 5]. To the best of our knowledge, rare studies have systematically described the prenatal diagnosis due to the limitations of phenotypic identification in prenatal practice. Here, we investigated the frequency of the microdeletions and microduplications associated with chromosome 16 in 2,414 fetuses who underwent prenatal CMA for different indications. Our data emphasize the value of CMA in prenatal diagnosis and the importance of long-term postnatal follow-up for fetuses with likely pathogenic aberrations.

## Results

**Fig. 1:**
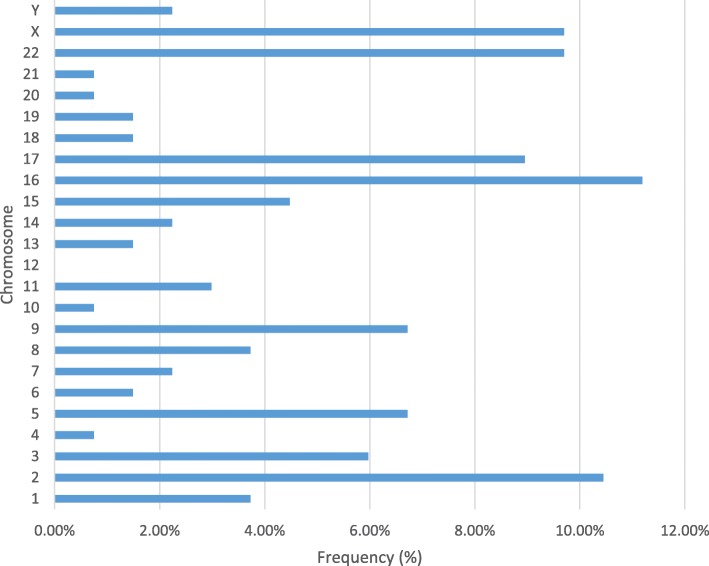
The frequency of 134 submicroscopic anomalies on each chromosome. All chromosomes except for chromosome 12 were involved. Among them, 11.1% (15/134) aberration derived from chromosome 16, which had the highest frequency, followed by chromosome 2, 22 and X

A total of 134 submicroscopic aberrations were identified in 117 (4.8%, 117/2,414) fetuses with normal karyotype or balanced structural aberration, including 51 cases of copy gain, 66 cases of copy loss, and 17 cases of loss of heterozygosity (LOH). Aberrations derived from chromosome 16, 11.1% (15/134), were observed with the highest frequency, followed by those of chromosome 2 and 22 (Fig. 1). Copy number variants (CNVs) of chromosome 16 ranged from 600 kb to 2.24 Mb in size (Table 1).

**Table 1:** Characterization of the 15 submicroscopic aberrations associated with chromosome 16 detected from 14 patients

Patients	Indications	Specimens	Karyotypes	SNP array results	Size	Inheritance	Clinical significance	Pregnancy outcome
1	Bilateral lateral ventricle dilatation	Amniotic fluid	46,XN	arr[GRCh37] 16p13.11 (15,058,820_16,309,046)× 3	1.25 Mb	Paternal	VOUS (likely benign)	Live born infant, Normal development at 8 months
2	Echogenic bowel; mild tricuspid regurgitation	Amniotic fluid	46,XN	arr[GRCh37] 16p13.11 (15,058,820_16,309,046)× 3	1.25 Mb	Unknown	VOUS	Live born infant, Normal development at 12 months
3	Echogenic bowel	Cord blood	46,XN	arr[GRCh37] 16p13.11 (15,171,146_16,309,046)×3	1.1 Mb	Maternal	VOUS (likely benign)	Live born infant, physical retardation
4	Urorectal Septum Malformation Sequence	Cord blood	46,XN	arr[GRCh37] 16p13.11 (15,325,072_16,272,403)×3	947 kb	Unknown	VOUS	TOP
5	AMA	Amniotic fluid	46,XN	arr[GRCh37] 16p13.11 (15,481,747_16,278,133)×3	796 kb	De novo	VOUS (likely pathogenic)	Live born infant, normal development at 14 months
6	Bilateral lateral ventricle dilatation; echogenic bowel	Cord blood	46,XN	arr[GRCh37] 16p13.11 (15,422,960_16,508,123)× 1	1.0 Mb	De novo	Pathogenic	TOP
7	Bemivertebra (L4)	Cord blood	46,XN	arr[GRCh37] 16p11.2 (29,428,531_30,190,029)× 1	761 kb	De novo	Pathogenic	TOP
8	Lateral ventricle dilatation	Cord blood	46,XN	arr[GRCh37] 16p11.2 (29,567,296_30,190,029)× 1	600 kb	De novo	Pathogenic	TOP
9	Hemivertebra (T12)	Cord blood	46,XN	arr[GRCh37] 16p11.2 (29,580,020_30,190,029)× 1	610 kb	De novo	Pathogenic	TOP
10	Hydrocephalus, ventricle dilatation	Cord blood	46,XN	arr[GRCh37] 16p11.2 (29,591,326_30,176,508)×1	585 kb	De novo	Pathogenic	TOP
11	Echogenic intracardiac focus	Amniotic fluid	46,XN	arr[GRCh37] 16p11.2 (32,024,388_33,800,323)×3	1.7 Mb	De novo	VOUS	Live born infant, normal development at 22 months
12	AMA	Amniotic fluid	46,XN	arr[GRCh37] 16p12.2 (21,740,199_22,718,351)×1	978 kb	Unknown	VOUS	Live born infant, normal development at 15 months
13	High risk for Down’s syndrome screening, Chromosomal abnormal child birth history: seq[GRCh37]dup(14) (q22.2q22.3) 1.65 Mb pat	Amniotic fluid	46,XN	arr[GRCh37] 16q23.3q24.1 (82,786,394_85,029,292)×1	2.24 Mb	Maternal	VOUS (likely benign)	Live born infant, normal development at 9 months
14	FGR, VSD, aortic stenosis, left kidney dysplasia or absence, Echogenic intracardiac focus	Amniotic fluid	46,XN	arr[GRCh37] 16q23.2q24.3 (79,800,878_90,146,366) hmz,16p13.3p12.3 (94,807_19,302,326) hmz	19.2 Mb	De novo	Pathogenic	TOP
					10.3 Mb	De novo	Pathogenic	

*AMA* advanced maternal age, *TOP* termination of the pregnancy, *FGR* fetal growth restriction, *VSD* ventricular septal defect

Cases 1–6 harbored CNVs of different size in chromosome locus 16p13.11. Cases 1 and 2 (boy infants) both showed a 1.25 Mb duplication in the same region of 16p13.11 and had normal phenotype during the follow-up. Case 1 inherited 827 kb of the 1.25 Mb deletion from a healthy father, with the additional repeat segment encompassing *NTAN1* (OMIM # 615367), a candidate schizophrenia gene. Ultrasonography in both cases showed abnormalities of soft markers: bilateral lateral ventricle dilatation for Case 1, echogenic bowel and mild tricuspid regurgitation for Case 2. Case 3 harbored a maternally inherited 1.1-Mb duplication. The mother had normal phenotype. The fetus showed ultrasound soft marker abnormality and was delivered at 37^+ 4^ weeks with a birth weight of 2.4 kg. However, at the follow-up after 12 months, the weight was 4.4 kg, which indicated physical retardation. In Case 4, a 947-kb duplication in the region of 16p13.11 was present in a fetus with Urorectal Septum Malformation Sequence (URSMS). Microduplications of 16p13.11 usually have incomplete penetrance and/or variable expression. They mainly manifest as cognitive impairment, behavioral disorders, congenital heart defects, and skeletal malformations, thus that CNV may not have been the reason for URSMS. In Case 5, a de novo 796-kb duplication in 16p13.11 was found in a fetus with normal ultrasound data. The infant showed normal development during 14-month follow-up. In the current study, we reported the hereditary 16p13.11 duplication as “likely benign”. In contrast with the five above cases, Case 6 harbored *a* de novo 1.0-Mb deletion in 16p13.11 region, associated with the 16p13.11 microdeletion syndrome. The fetus manifested with echogenic bowel and progressive bilateral ventriculomegaly, which could be caused by the CNVs. We classified it as a pathogenic variants and the parents opted for the termination of the pregnancy.

Four cases of de novo CNVs (Cases 7–10) represented the known clinical 16q11.2 microdeletion syndrome (OMIM #611913). They all had a ~ 600 kb deletion encompassing the critical region of a typical 16q11.2 microdeletion syndrome. Two fetuses showed congenital malformations involving the spine (Cases 7 and 9), whereas Cases 8 and 10 showed brain abnormalities. These pregnancies were terminated after counseling. Case 11 showed a de novo 1.7-Mb duplication in region 16p11.2, which was classified as variants of uncertain significance (VOUS) as it involved no OMIM genes and was not found in public CNV databases. Considering that there was only a single soft marker abnormality, the fetus had termed birth.

**Fig. 2:**
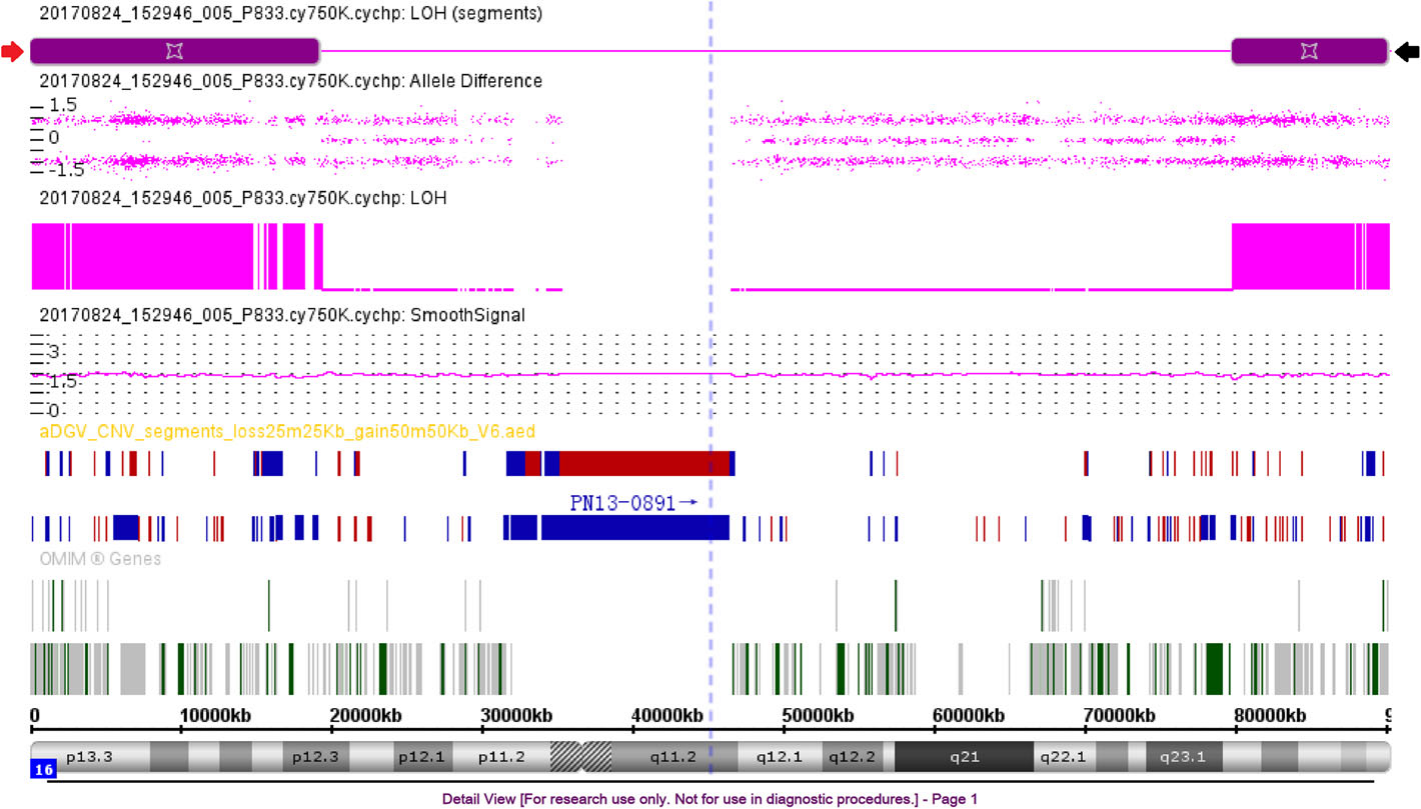
SNP array results for the individual of UPD(16). SNP array analysis revealed two copies of neutral LOH of 19.2 Mb in 16p13.3p12.3 (red arrow) and 10.3 Mb in 16q23.2q24.3 (black arrow) regions, respectively

Case 12 had a 978-kb deletion in region 16q12.2, containing four OMIM genes: *OTOA* (607038), *UQCRC2* (191329), *EEF2K* (606968), *CDR2* (117340). This deletion was reported as benign in DGV, but some cases with pathologies were described in DECIPHER. In addition, the deleted segment partly overlapped with the 16p11.2–12.2 deletion syndrome region, and it also contained a microdeletion of a susceptibility site for a neurodevelopmental disorder. The inheritance mode was unclear as the parents declined the analysis of their samples. Thus it was interpreted as “VOUS”, more likely pathogenic. Finally, an infant with normal phenotype was delivered and had normal development during 15 months of the follow-up. Case 13 harbored a 2.24-Mb deletion in region 16q23.3q24.1, involving 14 OMIM genes. Parental testing revealed maternal inheritance from healthy mother. Case 14 revealed two copies of neutral LOH of 19.2 Mb and 10.3 Mb in 16p13.3p12.3 and 16q23.2q24.3 regions, respectively (Fig. 2). A maternal isodisomic uniparental disomy (iUPD) was confirmed after trio analysis with the UPD tool.

## Discussion

The presence of segmental duplications or LCRs on chromosomes facilitates the occurrence of non-allelic homologous recombination during meiosis. It may result in microduplications and microdeletions [6, 7]. This is particularly evident in human chromosome 16, microduplications and microdeletions of which cause serious clinical syndromes. Here, we described 14 fetuses with segmental rearrangements mainly representing 16p11.2 microdeletion syndrome, 16p11.2–p12.2 microdeletion syndrome, 16p13.11 microdeletion syndrome, or 16p13.11 microduplication syndrome. It must pointed out that submicroscopic aberrations associated with chromosome 16 were most frequent of all submicroscopic aberrations detected in the present study, consistent with the results obtained by Cheng et al [8]. In some other studies, aberrations of chromosome 22 or X chromosome were more frequent than those of chromosome 16. Nonetheless, the frequency of submicroscopic changes associated with chromosome 16 is still relatively high [4, 5].

The 16p13.11 region contains nearly 14 known protein-coding genes. Unbalanced structural variation, deletions, and duplications occur most frequently in the short arm [9]. *NDE1* (nudE nuclear distribution gene E homolog 1) and *NTAN1* (N-terminal asparagine amidase) are the two genes that may be relevant to the neurocognitive phenotype. Loss or mutation of these genes has resulted in neurological manifestations in animal models, but the phenotypic consequences of gain are not that clear [10, 11]. Microdeletions of 16p13.11 have been associated with multiple phenotypic manifestations, including neurodevelopmental phenotypes such as autism, epilepsy, and non-CNS phenotypes, such as physical dysmorphisms and other congenital anomalies [12–15]. Evidence has accumulated that 16p13.11 microduplication may be associated with autism, schizophrenia, epilepsy, and attention-deficit hyperactivity disorder [15–17]. However, both types of aberrations were also detected in individuals with normal phenotype [11, 15]. This was interpreted by limited follow-up time. In our cohort, CNVs of 16p13.11 were most frequent (6/14). Five cases of 16p13.11 duplication were detected in fetuses with minor ultrasound abnormalities (*N* = 3), no ultrasound abnormalities (*N* = 1), and a fetus with Urorectal Septum Malformation Sequence (*N* = 1), with the latter case ending in the termination of pregnancy. Only one live born showed abnormal phenotype: physical retardation and speech delay during 14-month follow-up. Notably, the duplication in this case was inherited from a heathy mother. The lack of a phenotype in the mother may be attributed to incomplete penetrance that was reported in 10.6% of mutations associated with developmental delay [18].

16p11.2 microdeletion syndrome was first proposed as a risk factor for autism spectrum disorder in 2008, with a population prevalence of approximately 0.03% [19–21]. The phenotypic spectrum includes autism, developmental retardation, mental retardation, spinal deformity, and a range of neuropsychiatric developmental diseases. The phenotypic features vary according to the size and location of the deletion. In the present study, four cases of 16p11.2 microdeletion were all located in the 29 M–30.2 Mb region and belonged to Group 1 [22], which is the most common type, involving *HIRIP3*, *SEZ6L2*, *TBX6*, and other genes [23, 24]. Insufficient expression of *SEZ6L2* may be an important factor leading to speech delay and autism [25]. Mice homozygous for the loss-of-function mutation of *Tbx6* show irregular phenotypes, such as rib fusion, spine fusion, and vertebral body fusion, indicating that insufficient expression of *TBX6* in individuals with 16p11.2 microdeletion is likely responsible for spinal deformity [26, 27]. Studies have reported that the polymorphism of the *TBX6* gene is associated with hemivertebra and scoliosis in the Chinese Han population [28]; two in four cases in our study showed hemivertebra, consistent with the phenotype.

Ballif et al. established the identity of 16p11.2–p12.2 microdeletion syndrome by analyzing individuals with mental retardation, developmental delay, and common dysmorphic features [29]. It has a major difference from the 16p11.2 microdeletion syndrome, as it generally does not cause autism [30]. So far, almost all the patients share the same proximal breakpoint ~ 21.4 Mb and the distal break point is within ~ 28.5 Mb and ~ 30.1 Mb. Here, Case 12 had just a 978-Kb fragment overlapping with the 16p11.2–p12.2 microdeletion region, involving four OMIM genes, among them *OTOA* (607038) which is associated with hearing impairment, so it was classified as likely pathogenic.

UPD occurs when both members of a particular chromosome pair derive from the same parent and there is no contribution from the other parent. Case 14 was identified to have maternal iUPD(16), with the regions similar to those reported by Xie Yingjun et al. in a fetus with Fetal Growth Restriction (FGR). Case 14 fetus presented with FGR, congenital heart defect, and congenital renal dysplasia. UPD may cause clinical abnormalities through some common genetic mechanisms, including autosomal recessive disease, mosaicism, and imprinting. The regions of heterozygosity involved two genes associated with autosomal-recessive diseases, *CDT1*, located at 16q24.3, and *ALG1,* located at 16p13.3 [31, 32]. However, these genes are unlikely to be the causes of FGR. Seven imprinted genes, *SOX8*, *ZNF597*, *NAA60*, *SALL1*, *C16orf57*, *ACD*, and *FOXF1*, have been identified on chromosome 16. Among them, *ZNF597* located in 16p13.3 is expressed in the brain, leucocytes, and placenta [33]. It is maternally expressed, and its excessive expression in UPD(16) mat patients may affect placental development and cause growth failure [34, 35]. Thus, imprinting is a reasonable explanation for disease pathogenesis here.

There are many loci of susceptibility to neurodevelopmental disorders in chromosome 16 characterized by variable expression level and heterogeneity of clinical features [36]. Most fetuses do not have specific ultrasound findings in the uterus and often manifest only abnormal ultrasound soft markers. In such cases, a long period of follow-up is required for better prognosis; therefore, revealing information about these CNVs may be somewhat controversial. Most often, we reported such CNVs as “likely pathogenic”. In the current study, the majority of “likely pathogenic” cases had normal development, which confirmed that phenotypic consequences of many CNVs for the fetus are always uncertain and that insufficiently long follow-up may not predict the phenotype accurately.

## Conclusions

Submicroscopic aberrations on chromosome 16 are frequent findings, which emphasize the importance of CMA in prenatal diagnosis. These aberrations are mainly associated with the susceptibility to central nervous system disorders, and as they rarely cause specific ultrasound abnormalities, the long-term follow-up is needed to establish their pathogenicity. Prenatal diagnosis helps to ensure long-term follow-up of such children, which is critical for better understanding of the syndromic phenotypes of chromosome 16 microdeletion and microduplications.

## Materials and methods

### Patients and samples

This retrospective study reviewed 2414 consecutive patients who underwent invasive prenatal diagnostic testing between January 2016 and October 2018 at the prenatal diagnosis center of Fujian Maternal and Child Health Hospital, affiliated hospital of Fujian Medical University, China. The mean maternal age ranging from 17 to 47 years, and gestational age ranging from 13 to 33 weeks. The samples comprised specimens of chorionic villus (*n* = 25), amniotic fluid (*n* = 1,819), and fetal cord blood (*n* = 570). The referral indications included advanced maternal age, abnormal trimester screening, abnormal pregnancy history, ultrasound abnormality. The study was approved by the local Ethics Committee of the Fujian Maternal and Child Health Hospital. Genetic counseling was provided to the patients before prenatal testing and written informed consent was obtained from all of them.

### CMA platforms and data interpretation

CMA was performed with an Affymetrix CytoScan 750 K array (Affymetrix Inc., Santa Clara, CA, USA), which includes 200,000 probes for single nucleotide polymorphisms and 550,000 probes for copy number variations (CNVs) distributed across the entire human genome. The resolution for CNVs was ≥200 kb for deletions, ≥500 kb for duplications, and ≥ 10 Mb for the loss of heterozygosity (LOH). To analyze the results, Chromosome Analysis Suite software (Affymetrix) and human genome version GRCh37 (hg19) were used. All detected CNVs were compared with those listed in the following publically available databases: Database of Genomic Variants (DGV), Database of Chromosome Imbalance and Phenotype in Humans Using Ensemble Resources (DECIPHER), International Standards for Cytogenomic Arrays Consortium, and Online Mendelian Inheritance in Man (OMIM). Uniparental disomy (UPD) was reported based on the identification of the region of homozygosity (ROH) covering the entire chromosome (complete isodisomic uniparental disomy, complete iUPD), a single large (20 Mb) or multiple segments of ROH on a single chromosome (segmental iUPD). The family microarray data were processed to confirm maternal or paternal iUPD origin. They were classified using the UPD tool available at the following link: (http://upd-tl.com/upd.html).

The CNVs were classified according to the American College of Medical Genetics (ACMG) definitions [37]: pathogenic, benign, and variants of uncertain significance (VOUS) which can be further classified as likely pathogenic, likely benign, and CNVs of little or no relevant clinical information. Parental blood samples were collected and analyzed by CMA to provide more information if the aberration was determined to be potentially clinically significant.

### Conventional karyotyping

Conventional karyotyping consisted of cell culture and G-banded karyotyping was performed according to the standard protocols in our laboratory at the 320–500 bands level. The karyotype was determined according to the International System for Human Cytogenetic 2016 (ISCN 2016).

### Clinical follow-up

For all fetuses diagnosed with VOUS, likely pathogenic or pathogenic variants, parental DNA testing by SNP array was offered to define parental origin in order to further interpret the pathogenicity of fetal CNVs. The long-term follow-up was performed with a median infant age of 11 months through the patient’s medical record or telephone inquiry.

## Data Availability

The datasets used and/or analyzed during the current study are available from the corresponding author on reasonable request.

## Abbreviations

AMA: Advanced maternal age
CMA: Chromosomal microarray analysis
CNVs: Copy number variants
FGR: Fetal growth restriction
iUPD: Isodisomic uniparental disomy
LCRs: Low copy repeats
LOH: Loss of heterozygosity
TOP: Termination of the pregnancy
UPD: Uniparental disomy
VSD: Ventricular septal defect
